# Simulation and optimization of rectenna systems

**DOI:** 10.1038/s41598-023-44401-2

**Published:** 2023-10-10

**Authors:** Melad M. Olaimat, Vahid Nayyeri, Omar M. Ramahi

**Affiliations:** 1 Department of Renewable Energy, Al al-Bayt University, Al Mafraq, Jordan; 2 School of Advanced Technologies, Iran University of Science and Technology, Tehran, Iran; 3 Department of Electrical and Computer Engineering, University of Waterloo, Waterloo, ON N2L 3G1 Canada

**Keywords:** Devices for energy harvesting, Electrical and electronic engineering

## Abstract

In this paper, we present an entirely simulation-based method to predict the performance of a complete rectenna system that includes all its components: the receiving antenna, the matching circuits between the antenna and the rectification circuit, and the load circuit. Whereas previous efforts to predict the performance of a rectenna system subdivided the system into the antenna part (radiation to AC power conversion) and the circuit part (AC power to DC power conversion), and made assumptions about the performance of the non-linear part of the rectenna based on a specified power level and frequency, in this method, the radiation part of the system is incorporated into the simulation by using Thevenin theorem. The method proposed in this work enables the rectenna designer to predict the performance of the complete rectenna system, at the design stage, for variation in the incident field’s power density, angle of incidence, and operating frequency. Such performance prediction was not available before. Furthermore, the proposed method enables the rectenna designers to optimize the entire system over a portion of or the entire range of the operating frequency. Experimental results are provided to demonstrate the accuracy of the method.

## Introduction

Driven by unprecedented demands on energy, the need for new paradigms that produce clean and affordable energy becomes increasingly desired. Recently, there has been intensive research to investigate the ability of rectenna systems to harvest the energy from electromagnetic (EM) radiation (for a sample of recent works, please see^1–15^ and references therein). To harvest EM radiation, a rectenna is used, which consists of an antenna or a surface that captures the EM radiation, a rectifying circuit, and a DC load circuit. The antenna acts as a transducer that converts the incoming EM radiation into an electrical signal. The rectifying circuit, which consists of a single or multiple diodes and a matching circuit, converts the AC power into DC power.

The rectenna system can be used in a power transmission link (point-to-point), where the design constraints of the rectenna can be tailored to the nature of the largely known (or predictable) incident field, including its frequency, polarization and power density (see^16^ as an example), or can be used to harvest ambient EM radiation over specific frequency band (see^17,18^ as an example). In the latter application, where the rectenna acts as an energy scavenger, since the incoming radiation is highly unpredictable in terms of frequency bandwidth, polarization and power density, the design of the rectenna can be challenging.

Harvesting the ambient EM radiation was investigated in urban and semi-urban areas. For instance, in^17^, it was demonstrated that an ambient EM wave with power density of – 25 dBm/cm$^2$ can be harvested with 40% efficiency using a single band rectenna, and an ambient wave with power density of – 29 dBm/cm$^2$ can be harvested with a multi-band rectenna system. To evaluate the efficiency of a system with fluctuating levels of available power, in^17^, the efficiency was calculated using energy instead of power.

One of the key challenges when designing a rectenna system is the non-linearity of the rectifying circuit. Due to the diode’s non-linearity, the input impedance of the rectifying circuit is sensitive to the operating frequency and power level. Due to the non-uniform input impedance of antennas with frequency, any change in the operating frequency or in the angle of incidence would result in a change in the available power at the terminals of the antenna, which feed the rectification circuit. Consequently, to properly simulate the behaviour of the complete rectenna, all variations in the power level, the input impedance, and the frequency should be taken into consideration. This challenge is demonstrated by numerous previous works. For example, Lu et al. proposed a configurable rectenna system that operates at two different frequencies where the efficiency of the system was evaluated for different values of power; however, an agreement between the simulation and measurement results was obtained only at a specific power level^19^. Nie et al. obtained good agreement between the simulation results and measurements only over a narrow-band close to the operating frequency; however, outside this narrow-band, the simulation results deviated from the measurement results significantly^20^. In other works, the variation of the input impedance of the antenna with frequency was taken into account, however, the input power was assumed constant with with frequency^21–23^. In these works the simulation procedure gives accurate prediction of the rectenna performance over a very narrow frequency range; however, for other frequencies, the prediction would deviate appreciably from the actual performance. In^24^, the Co-simulation was utilized to import the S-parameters of an antenna from an EM-based simulator to a circuit-based simulator, where the S1P file was utilized to incorporate the characteristic of the antenna’s input impedance versus frequency in the rectifying circuit’s input port. The fluctuation of the antenna’s input impedance with frequency was taken into consideration, but still the input power was assumed constant.

To achieve high rectenna efficiency, in^25,26^, the input power levels were into two ranges: a low range and a high range. Then two circuits were designed: one for the high range and a second one for the low range. The two circuits were then combined to give the final rectenna system. While these works claimed that the input voltage depends on the input power, no explanation of the relationship between the input voltage and the received power was provided. Moreover, the effect of the variation of the voltage with frequency was not analyzed.

In the literature, some studies used the Thevenin and Norton equivalent to replace the antenna part of the circuit. Some studies only used the equivalent circuit at a single frequency, limiting its applicability to a relatively small frequency range^22,23^. Other studies have recommended using the reciprocity theory to determine the open circuit voltage, however the deviation between the measured and simulated values was significant^27–29^ (for example, see the discrepancy between the simulation and measurement results in^28^). Using commercial EM simulators to numerically compute the open-circuit voltages of the receiving antenna for energy source in the near-field of the receiving antenna was suggested in^28^ however, without any demonstration of its applicability.

In this work, we present a method by which the entire rectenna system can be fully simulated while taking the variation of the incident power density, the input impedance of the antenna, the impedance of the rectification circuit, and frequency into account^30^. Specifically, the simulation presented would allow for the output DC power to be accurately predicted for any level of available electric field intensity at the antenna and over a range of frequencies. This can be achieved by utilizing a commercial EM simulator to mimic the behavior of the antenna in receiving mode and exporting all the required parameters to a circuit simulator.

The rest of the paper is ordered as follows: section two presents the theory underlying the present method. Section "Theory" presents the theory behind our method. Section "Application" demonstrates the application of the method to a rectenna employing a triangular patch antenna. Section "Optimization of rectenna" gives a full validation of the method using measurements. Section "Validation" provides a summary of the main contributions.

**Figure 1:**
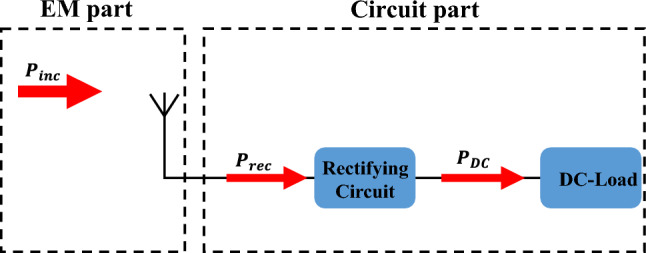
Basic components of a generic rectenna system comprising a receiving antenna, a rectification circuit (may include matching circuits on both ends), and a DC load.

## Theory

Figure 1 shows a diagram of the basic components of a generic complete rectenna system. As depicted in the Figure, the rectenna includes the antenna that receives the incoming EM radiation, and the circuit part comprises a rectifying circuit and a DC load. In Fig. 1, $P_{inc}$ represents the incoming power density at the antenna from an external electromagnetic source, $P_{rec}$ is the power received at the terminals of the antenna, and $P_{dc}$ is the output DC power. There are two efficiencies that combine to give the full rectenna efficiency. The first is the radiation to AC conversion efficiency defined as the ratio between $P_{rec}$ and $P_{inc}$:

1 $$\begin{aligned} \eta _{rad-ac}=\frac{P_{rec}}{P_{inc}}, \end{aligned}$$

where $P_{inc}$ is the power available at the antenna, and $P_{rec}$ is the power available at the terminals of the antenna (all are time-average power). The second is the AC to DC conversion efficiency, which is defined as

2 $$\begin{aligned} \eta _{ac-dc}=\frac{P_{dc}}{P_{rec}} =\frac{(V_{dc}^{2} / R_{L})}{P_{rec}} \end{aligned}$$

where $R_L$ is the load resistance, and $V_{dc}$ is the voltage across $R_L$. The full rectenna efficiency is then given by

3 $$\begin{aligned} \eta =\eta _{rad-ac} \times \eta _{ac-dc} \end{aligned}$$

**Figure 2:**
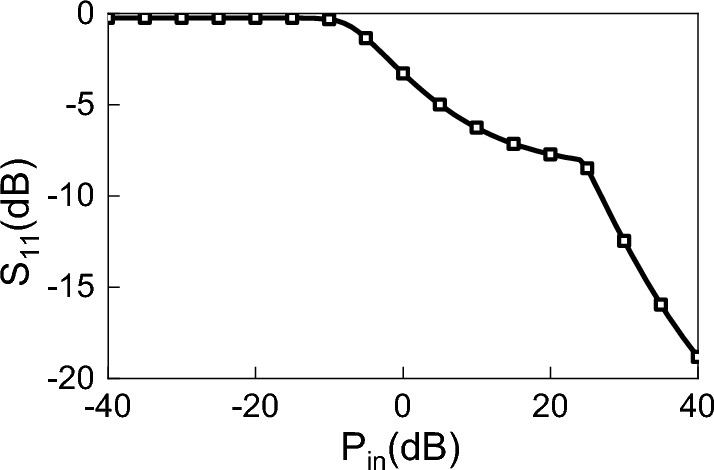
Variation of the reflection coefficient of the diode with input power at 2 GHz.

To maximize power transfer from the antenna terminals to the input of the diode, a matching circuit is placed between the antenna and the diode or the rectification circuit (such a matching circuit may be considered as part of the rectification circuit). Due to the non-linearity of the rectification circuit, its input impedance is sensitive to variations in the input power. This variation is a major challenge that prevented full characterization of the rectenna system over the operating frequency range of the rectenna. To demonstrate the sensitivity of the rectification circuit’s input impedance to the input power, we simulated the reflection coefficient of an HSMS-2860 diode (chosen as an example of the rectification circuit) for different levels of power, using the Advance Design Simulator (ADS) software^31^. Figure 2 shows the reflection coefficient of the diode for different values of power at 2 GHz when the diode is terminated by a 50 Ohm load. We observe that the reflection coefficient decreases with increasing input power. The wide variation of the reflection coefficient with the input power demonstrates the need to incorporate the power profile at the antenna terminals into the simulation of the entire rectenna system. If the power profile was not taken into account, the variation of the power level at the input of the rectification circuit would result in a significant discrepancy between the simulation’s results and the measurement’s results as has been demonstrated in previous works^21–23^.

The intuitive brute-force approach to incorporate all rectenna components into the simulation process entails combining the full-wave 3D model of the antenna and the circuit models of the rectification circuit (its linear and non-linear parts) in such a manner that the frequency bandwidth of the simulation stretches from the operating frequency of the antenna to DC while including all harmonics arising from the non-linear rectification circuit. Such an approach may not be impossible but would be highly challenging and most likely very computationally intensive. Another possibility would be to incorporate the V–I characteristics of the non-linear circuit into a full-wave time-domain simulator such as the finite-difference time-domain (FDTD) method (see^32^ as an example where diodes were incorporated into the FDTD method). Such an approach has significant challenges and limitations, which may require lumping the entire circuit (the linear and non-linear components) as a single load terminating the antenna (while occupying a single FDTD cell). Even if workable, such an approach may be suited for simulating electromagnetic scattering problems rather than simulating rectenna systems, particularly because the DC power at a specific load is desired, which precludes lumping all circuits into one element having a specific I–V characteristic. Additionally, the three-dimensional full-wave model may not be suitable for the optimization of rectification circuits to achieve maximum power transfer to the load.

**Figure 3:**
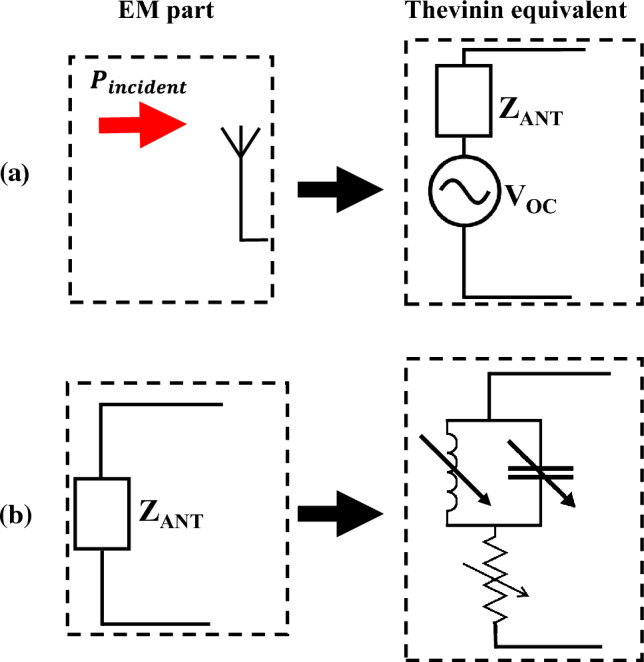
Equivalent circuit. (**a**) Thevenin equivalent circuit of an antenna. (**b**) Representation of the input impedance of the antenna.

Nevertheless, while time-domain simulation methods may be the most suitable “brute-force” method to simulate the performance of the entire rectenna system, the convergence of time-domain methods depends strongly on the time step, which can significantly increase the computational cost. Here we need to emphasize that the modeling approach needs to keep in mind an important objective when simulating rectenna systems, which is maximizing energy transfer from the antenna to a DC load. Therefore, a highly useful simulation strategy should allow for the optimization of the matching and rectification circuits to achieve the highest output DC power.

Instead of using a brute-force approach, our simulation methodology calls for modeling the antenna part as a circuit that is fully independent in its characteristics from the remaining parts of the rectenna system. This can be accomplished by using Thevenin’s theory. According to Thevenin theorem, a linear circuit can be replaced with an equivalent circuit consisting of an independent voltage source in series with an impedance as shown in Fig. 3a. The Thevenin equivalent circuit provides all the required information about the antenna. It is important to emphasize that the equivalent circuit of the antenna obtained using Thevenin theory is not affected by the rectenna circuit that connects the antenna to the load. In Fig. 3a, $V_{oc}$ represents the open-circuit voltage generated between the receiving antenna’s terminals when the terminals are left open and disconnected from the rectenna circuit. $Z_{ant}$ is the internal impedance of the receiving antenna.

While the antenna acts as an independent source as far as the rectenna circuit is concerned, if the source of radiation (such as a transmitter antenna) were included in the simulation of the antenna, the antenna would then be considered a dependent source. However, the goal of our simulation is to provide a prediction of the DC power for a range of incident power density and incident field polarization profiles. This would then provides, at the design stage, a full prediction of the DC power for an incident field with specific power density and polarization. Therefore, $V_{oc}$ of the Thevenin equivalent circuit would correspond to an incident field with a specific power density and polarization.

To extract $V_{oc}$, we illuminated the antenna by a plane wave of fixed power density and fixed incident angle and polarization. The CST Microwave Studio simulator was used to simulate the antenna^33^. While $V_{oc}$ is dependent on the characteristics of the incident field, $Z_{ant}$, however, is independent of these characteristics and can be found by either feeding the antenna with a source and then taking the ratio of the feed voltage to the input current, or by calculating the current in a short circuit $I_{sc}$ and then taking the ratio $V_{oc}$/$I_{sc}$ which will be equal to $Z_{ant}$. While it may not be intuitive, the ratio $V_{oc}$/$I_{sc}$ will always be equal to $Z_{ant}$ irrespective of the characteristics of the incident field.

The extracted values of $V_{oc}$ are stored in data access component (DAC) in ADS. DAC acts as an accessible database that stores a table of data and allows the simulator to recall the required value corresponding to the index value. Using the voltage source connected with DAC enables the voltage source to mimic the variation of the voltage across the terminals of the antenna.

To simulate the variation of $Z_{ant}$ with frequency, we designed the equivalent circuit illustrated in Fig. 3b. The equivalent circuit comprises an inductor and a capacitor connected in parallel and connected in series with a resistor. The values of the real part of $Z_{ant}$ are assigned to the resistor (from the DAC data). The values of the imaginary part of $Z_{ant}$ are used to calculate the corresponding value of the capacitor or inductor.

**Figure 4:**
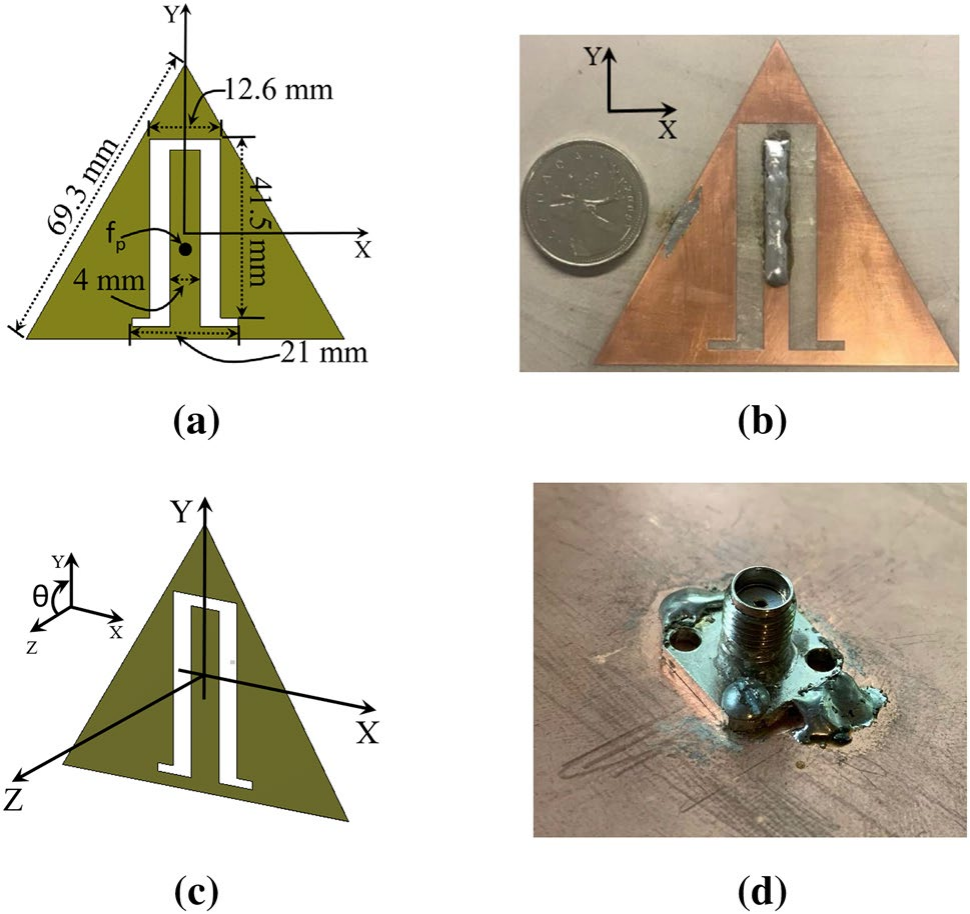
(**a**) Top view of the TPA with its dimensions. (**b**) The fabricated TPA. (**c**) Coordinate system used showing the angle $\theta$ of the incident field. (**d**) The fabricated feeding port of the TPA.

**Figure 5:**
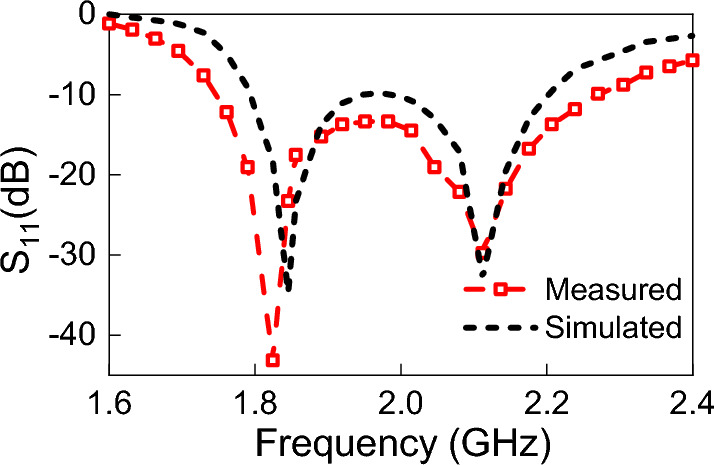
Simulated and measured reflection coefficient, S$_{11}$, of the proposed TPA.

## Application

### Design of the antenna

In this section, we demonstrate the applicability of our method to a rectenna system employing a practical antenna. As a receiving antenna, and without loss of generality, we employed a triangular patch antenna (TPA), which consists of a metallic radiating patch printed on a dielectric substrate and backed by a ground plane. The antenna resonant frequency is given by:

4 $$\begin{aligned} f_{m, n}=\frac{2 c}{3 a \sqrt{\varepsilon _{r}}} \sqrt{m^{2}+m n+n^{2}} \end{aligned}$$

where c is the velocity of light in free space, a is the side length (see Fig. 4), *m* and *n* are integers which are never zero simultaneously ($f_{1,0}$ is the fundamental frequency), and $\varepsilon _{r}$ is the dielectric constant of the substrate.

Here we developed a TPA to operate over the 1.8 to 2.2 GHz frequency range utilizing the optimization in the CST microwave studio simulator. Figure 4 shows the TPA with its dimensions and the angle reference for the incident field. As depicted in the Figure, the patch is based on an equilateral triangular design loaded with slots in the xy-plane. Due to embedding slots on the patch with a proper shape and size, two adjacent resonant modes merge together to form the bandwidth of the TPA. Moreover, introducing the slots reduces the large inductive reactance, consequently, impedance matching becomes easier over the frequency bandwidth.

**Figure 6:**
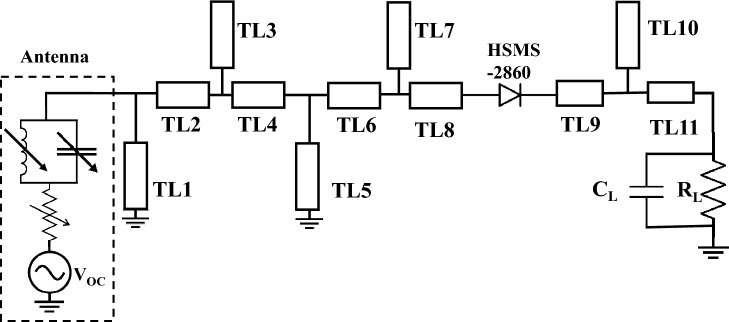
Schematic diagram of the rectifying circuit with the incorporated equivalent circuit of the antenna.

**Figure 7:**
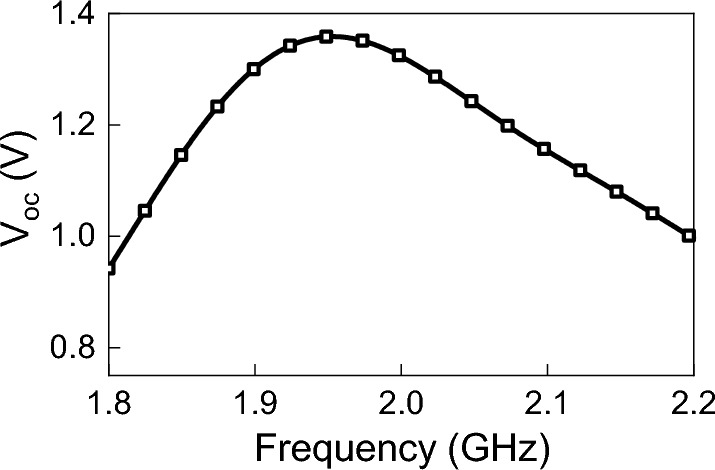
Simulated $V_{oc}$ for an incident plane wave having power density of 1 W/m$^2$, polarized in the y-direction and incident at an angle $\theta _{in}$=0.

**Table 1:** Lengths of the transmission lines segments used in the optimized rectification circuit.

TLn	Length (mm)	TLn	Length (mm)
TL1	14	TL7	2
TL2	5	TL8	2
TL3	6.6	TL9	2.2
TL4	6.2	TL10	2.2
TL5	5	TL11	10
TL6	9		

The design of the TPA comprises a stack of three substrate layers sandwiched by the ground and the patch. The layers thicknesses from the top are; 1.27 mm, 11.71 mm and 1.52 mm. Whereas the layers’ dielectric constants from the top are 2, 1 and 3.66. The TPA was fed by a coaxial cable located at 26.4 mm from the base of the triangle as illustrated in Fig. 4a. Figure 4d shows the realized SMA connector.

Figure 5 shows the simulated and measured reflection coefficient (S11) of the TPA. Figure 5 shows good agreement between the simulated and measured reflection coefficients of the TPA. The simulated reflection coefficient was below – 10 dB within the frequency range of 1.796 to 2.2 GHz, whereas the measurement results show a reflection coefficient of less than – 10 dB for the frequencies ranging from 1.75 to 2.26 GHz. For emphasis, we note that this antenna was chosen without loss of generality, and only for the purpose of the application of the method presented in this work.

### Design of the rectifying circuit

To design the rectifying circuit, we connected the Thevenin equivalent circuit of the TPA with the circuit shown in Fig. 6. In ADS, we utilized the DAC components to control the values of each component of the antenna’s equivalent circuit. To account for the circuit’s non-linearity, the harmonic balance controller was used in our simulation with the first five harmonics. Although increasing the harmonic order improves accuracy, the power content after the fifth harmonic is negligible. In fact, we conducted the simulation including higher order harmonics without noticing any significant change in the results.

As illustrated in Fig. 6, the circuit consists of HSMS-2860 Schottky diode^34^, 11 segments of transmission lines printed on the top of a 1.27 mm thick Roger 3006 dielectric substrate having a loss tangent of tan $\delta$ = 0.0027 and a dielectric constant of $\epsilon _r$ = 6.15, and a DC load. To achieve maximum output DC power, the widths of transmission lines’ segments were fixed to 1.8 mm. The lengths of segments and the DC load were optimized using the gradient optimization feature in ADS. The lengths of the segments of the optimized circuit are shown in Table 1. The optimum load was found to be a parallel combination of 100 pF capacitance and a resistance of 700 Ohm.

It is important to note that the optimized circuit was based on an incident field with specific characteristics in terms of polarization, incident angle, and power density. For wireless power transfer applications where the incident field characteristics are known, the optimized rectification circuit just described will deliver maximum power to the optimized load. However, for energy harvesting of incoming radiation without any known characteristics (aside from the frequency range), then the field-specific optimized rectification circuit may not be optimal over the possible variance in the characteristics of the incident field. However, we emphasize that the primary objective of this work is to enable full characterization (simulation) of the rectenna system irrespective of the type of rectification circuit used and without any fabrication of the circuit and the antenna.

**Figure 8:**
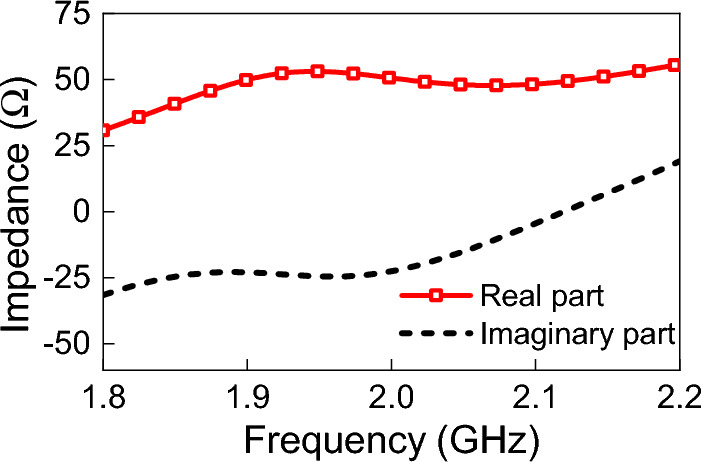
Simulated impedance of TPA obtained directly from CST.

Using the time-domain solver in CST, the antenna was illuminated by a linearly-polarized plane wave (y-polarization; see Fig. 4). The power density of the plane wave was 1 W/m$^2$ and the angle of incidence ($\theta$) was zero degrees (i.e. the k vector is coming from the positive z-direction. Then, $V_{oc}$ was extracted from the full-wave simulation of the TPA (when the antenna’s terminals were open-circuited), and is shown in Fig. 7. Figure 8 shows the simulated real and imaginary part of the TPA’s impedance.

**Figure 9:**
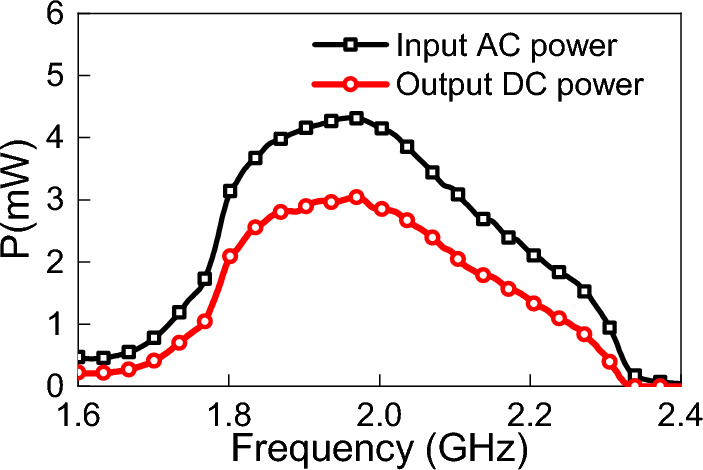
Simulated input and output power in mW.

**Figure 10:**
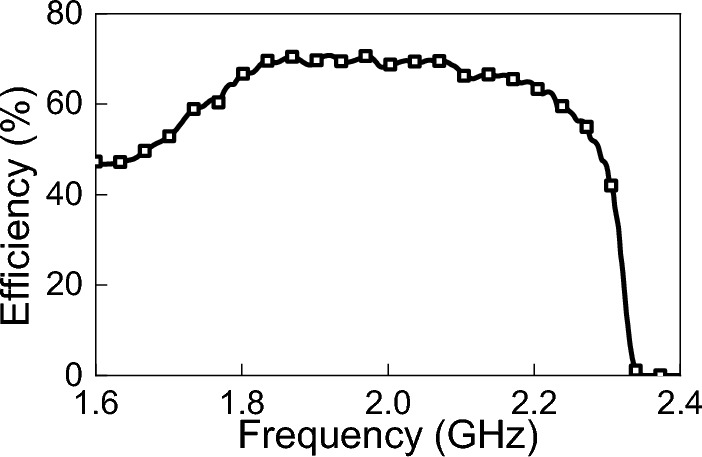
Power efficiency ($\eta _{ac-dc}$) of the rectifying circuit.

The extracted parameters are processed and loaded to the source as explained in the Theory section. Using ADS, the received AC power, $P_{rec}$, and the output DC power, $P_{dc}$, of the rectenna system were obtained (ref to Fig. 6). Figure 9 shows the simulation results of $P_{rec}$ and $P_{dc}$. Figure 10 shows the efficiency of the rectifying circuit. The efficiency of the rectifying circuit is greater than 65% over the operating range, with a maximum of 70% at 1.92 GHz.

## Optimization of rectenna

This section demonstrates how the proposed method can be utilized to optimize the design over any sub-band or the entire operating band. To enhance the overall efficiency of a rectenna system, especially in energy harvesting applications, we may need to improve the design performance within a certain frequency band where the energy concentration is relatively high. Having the entire data about the antenna, we were able to optimize the design illustrated in Fig. 6 over the required sub-band. Particularly, we divided the entire operating band ($R_T$) into four equally sub-bands, namely $R_1$ (1.8–1.9 GHz), $R_2$ (1.9–2 GHz), $R_3$ (2–2.1 GHz) and $R_4$ (2.1–2.2 GHz). To optimize the design over each sub-band, we adjusted the operating frequency in the harmonic balance simulation controller according to the needed sub-band and ran the optimization tool in ADS. The optimum parameters obtained for each sub-band and the whole band are depicted in Table 2.

**Table 2:** Parameters of the rectification circuits optimized over various bands (R).

Parameter	$R_T$	$R_1$	$R_2$	$R_3$	$R_4$
TL1 (mm)	14	12.8	12	16.7	19.2
TL2 (mm)	5	5.8	8.5	7	3.1
TL3 (mm)	6.6	8.1	6.6	5.9	5.6
TL4 (mm)	6.2	5.2	6.4	5.8	5.8
TL5 (mm)	5	2.9	3	3.2	2.7
TL6 (mm)	9	11	9.8	8.7	8.6
TL7 (mm)	2	2	2	2	2
TL8 (mm)	2	2	2	2	2
TL9 (mm)	2.2	3	3.9	3.4	2
TL10 (mm)	2.2	2	2	2	2
TL11 (mm)	10	12.4	10.5	10	10.1
$R_L$ ($\Omega$)	700	2555	2048	2254	2498
$C_L$ (pF)	100	400	338	232	334

**Figure 11:**
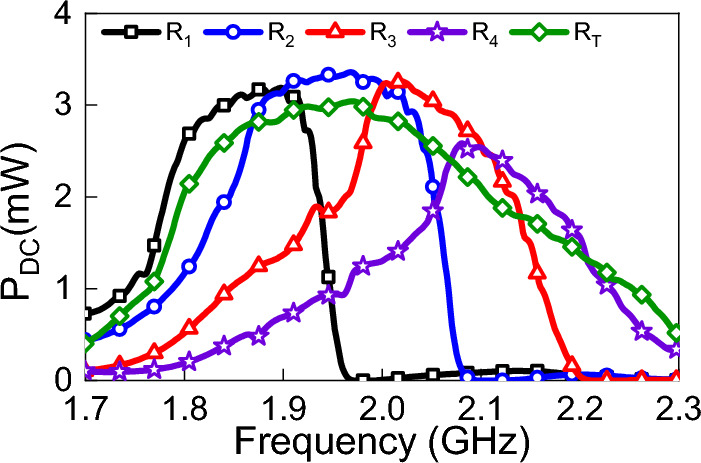
Simulated output power in mW optimized at various sub-bands.

Figure 11 shows the output DC power for the designs that are optimized at different sub-bands and for the design that is optimized at the entire range of frequency. As illustrated in the Figure, the proposed method enables us to improve the output DC power at required frequency ranges. On the other hand, the results obtained in Fig.  11 prove that the lack of data about the entire range limits our ability to optimize the system over the full range. In fact, without the availability of voltage values at all frequencies, optimizing the design across the entire band is impossible.

**Figure 12:**
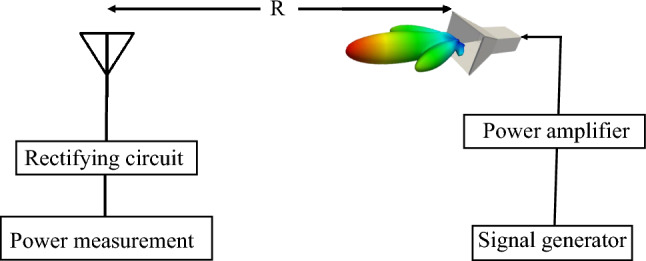
Measurements setup; the transmitting side is a horn antenna connected to a power amplifier and signal generator and the receiving side is a TPA connected to the rectifying circuit.

**Figure 13:**
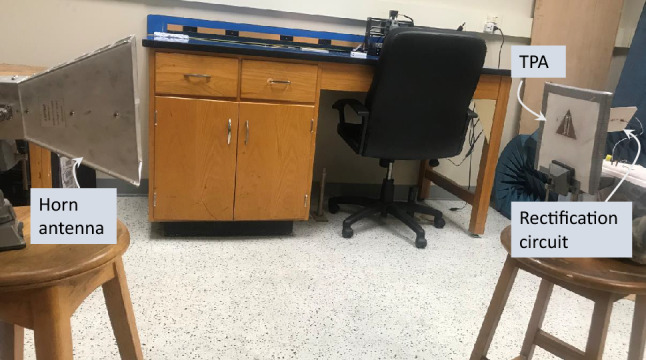
Laboratory measurement’s setup.

## Validation

The primary objective of the method presented in this work is to enable full simulation of the rectenna such that the designer can predict the accurate performance of the rectenna over the specified frequency range and for any angle of incidence and polarization. Additionally, the method allows for evaluating the performance of any rectenna system designed by a third party, without resorting to the measurements. To demonstrate the accuracy of our method, we present a comparison between the simulation results obtained by our method and measurements. To this end, a complete rectenna was designed, fabricated, and the DC output voltage was measured. The schematic for the experiment’s setup is shown in Fig. 12. Figure 13 shows the laboratory setup. The radiation source comprised a signal generator, a power amplifier, and a horn antenna. The receiving side includes the rectenna terminated by a capacitor in parallel with a load resistor. To mimic the simulation setup, we adjusted the output of the signal generator such that the power density at the rectenna was 1 W/m$^2$. The power density S at the rectenna can be calculated by^35^:

5 $$\begin{aligned} S=\left( \frac{G_{A} G_{T}(f) P_{T}}{4 \pi R^{2}}\right) \end{aligned}$$

where, $G_{A}$ is the amplifier gain, $G_T(f)$ is the gain of the horn antenna at different frequencies obtained from the datasheet, and $P_{T}$ is the output of the signal generator. The distance between the transmitter and receiver, R, is 1 m.

**Figure 14:**
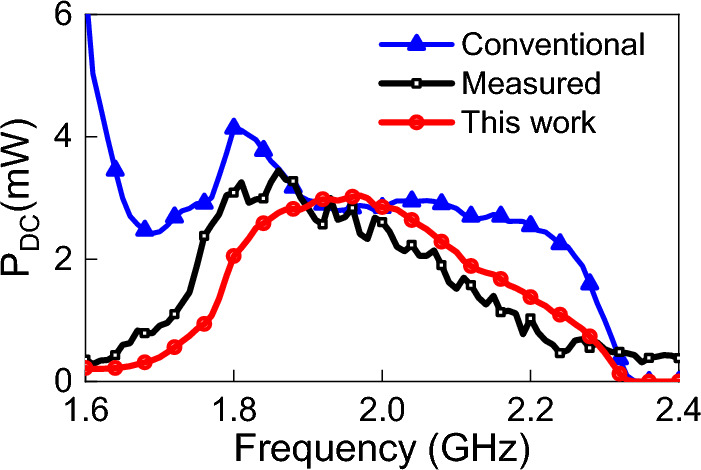
Simulated and measured output DC power. The incident field polarization was fixed (y-direction), the incident angle, $\theta$, was fixed at zero degrees (see Fig. 4), and the power density of the incident field was fixed at 1 W/m$^2$.

In order to measure the output DC power of the system, we terminated the circuit with the same load arrived at earlier using gradient optimization in ADS (a capacitor of 100 pF in parallel with a 700 Ohm resistor). Then the frequency was swept from 1.6 to 2.4 GHz while measuring the DC voltage across the load using a voltmeter. The DC output power was calculated as P$_{dc}$ = V$_{L}^2$/R$_L$, where V$_{L}$ is voltage across the load R$_L$. The results obtained from the measurements and simulations are shown in Fig. 14. We observe good agreement between the measurements and the full-rectenna simulation.

It can be observed from Fig. 14 that the measured DC voltage has a fluctuation that is more pronounced at higher frequencies. This is because the measurements were done in our laboratory to emulate a realistic scenario (rather than in perfect free space, or in an anechoic chamber). Therefore, it is highly probable that the fluctuations in the DC power plot is the result of multipath signals. In fact, it can be observed that the fluctuations increase with decreasing wavelength.

**Figure 15:**
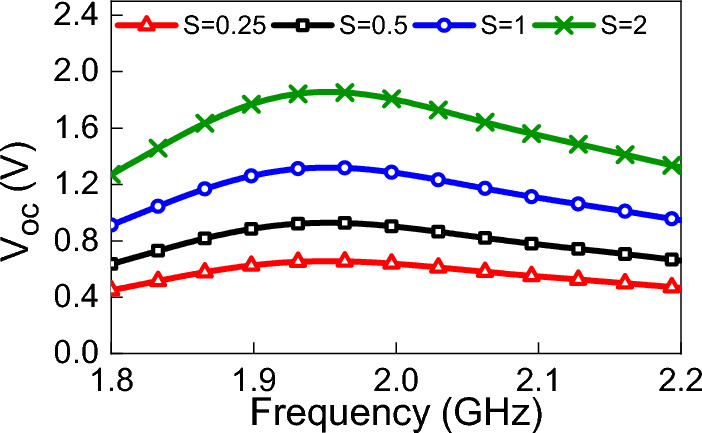
Simulated $V_{oc}$ for different power densities of the incident field. The incident field polarization was fixed (y-direction) and the incident angle, $\theta$, was fixed at zero degrees (see Fig. 4).

**Figure 16:**
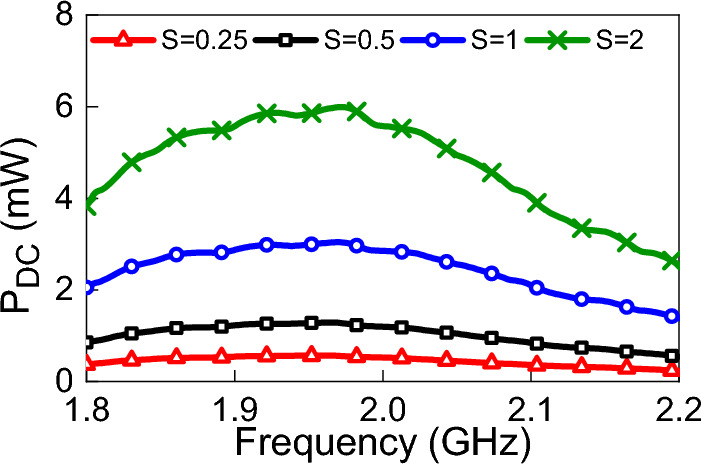
Simulated $P_{dc}$ for different power densities of the incident field. The incident field polarization was fixed (y-direction) and the incident angle, $\theta$, was fixed at zero degrees (see Fig. 4).

In addition to demonstrating the accuracy of our method, we provide a full rectenna performance comparison between the method presented in this work and the conventional method, which is expected to provide an accurate prediction of the DC output power over only a narrow range of frequency and input power (to the rectification circuit) specified during the rectenna design stage. In the conventional method adopted by previous works, instead of using a database to change the input voltage with frequency, the maximum input voltage was selected and fixed to provide the circuit with maximum power level at all frequencies. (The maximum power level was obtained at 1.95 GHz while the antenna was impedance-matched). From Fig. 14, we observe, as expected, that the simulation results obtained from the conventional method provide good agreement with measurements only over a very narrow frequency range around the 1.95 GHz; however, appreciable deviation from measurements is observed at other frequencies, especially over the 1.6 to 1.9 GHz and 2.1 to 2.3 GHz frequency bands.

**Figure 17:**
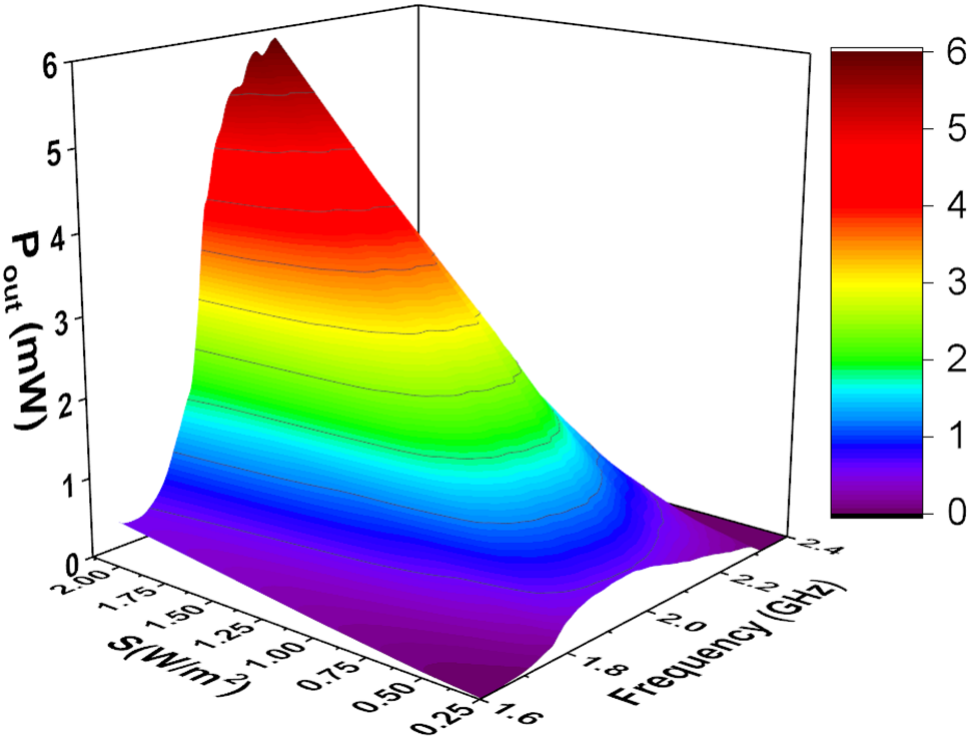
Simulated $P_{dc}$ for different power densities of the incident field and different frequencies. The incident field polarization was fixed (y-direction) and the incident angle, $\theta$, was fixed at zero degrees (see Fig. 4).

The available power density is an important factor that has an impact on the operation of the overall system. The power density may change due to many reasons such as, but not limited to, the existence of obstacles that block the coming wave, reflections from the surrounding environment, and weather conditions. To study the effects of changing the power density on the rectenna system, the rectenna was illuminated by a plane wave with different values of power densities. The polarization of the linear plane wave was in the y-direction as shown in Fig. 4, and the angle of incidence was set to zero (normal incidence from the positive z-direction). Figure 15 shows the variation of $V_{oc}$ for different values of power densities (four power density values of 0.25 W/m$^2$, 0.5 W/m$^2$, 1 W/m$^2$, and 2 W/m$^2$ were considered). Figure 16 shows the effect of changing the power density of the incident field on the output DC power. The non-linear behavior of the rectification circuit is highly visible in the results shown in Fig.  16. Utilizing the proposed method, we were able to produce a 3D Figure that simultaneously displays the fluctuation of the output DC power with frequency and the available power density, as illustrated in Fig. 17. The 3D figure enables the designer to determine the optimum operating frequency and the optimum power density.

**Figure 18:**
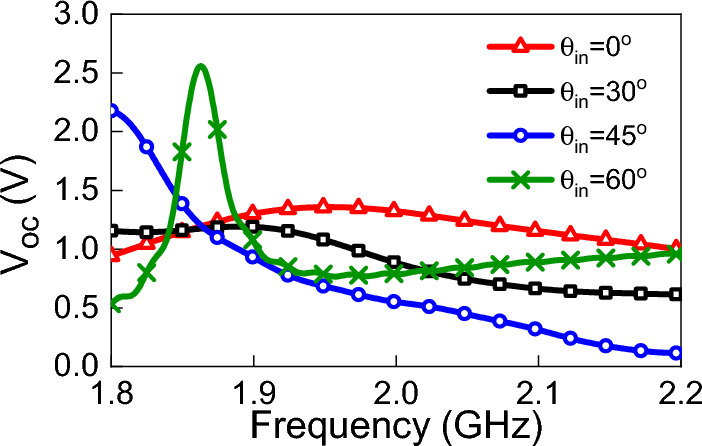
Simulated $V_{oc}$ values for different angles of incidence. The power density of the incident field was kept constant at 1 W/m$^2$.

**Figure 19:**
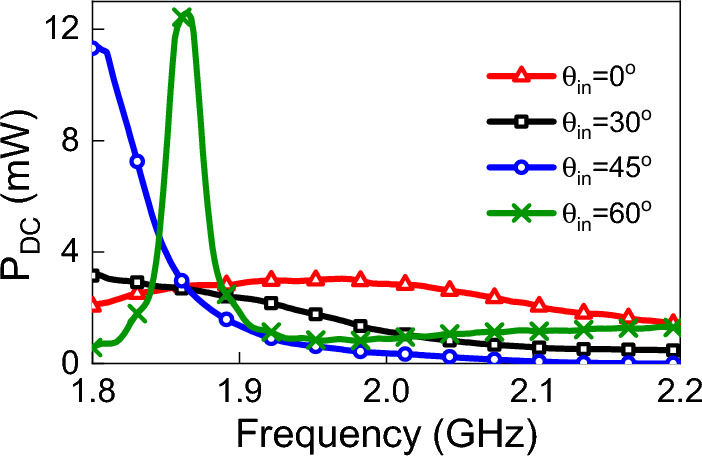
Simulated $P_{dc}$ for different angles of incidence. The power density of the incident field was kept constant at 1 W/m$^2$.

**Figure 20:**
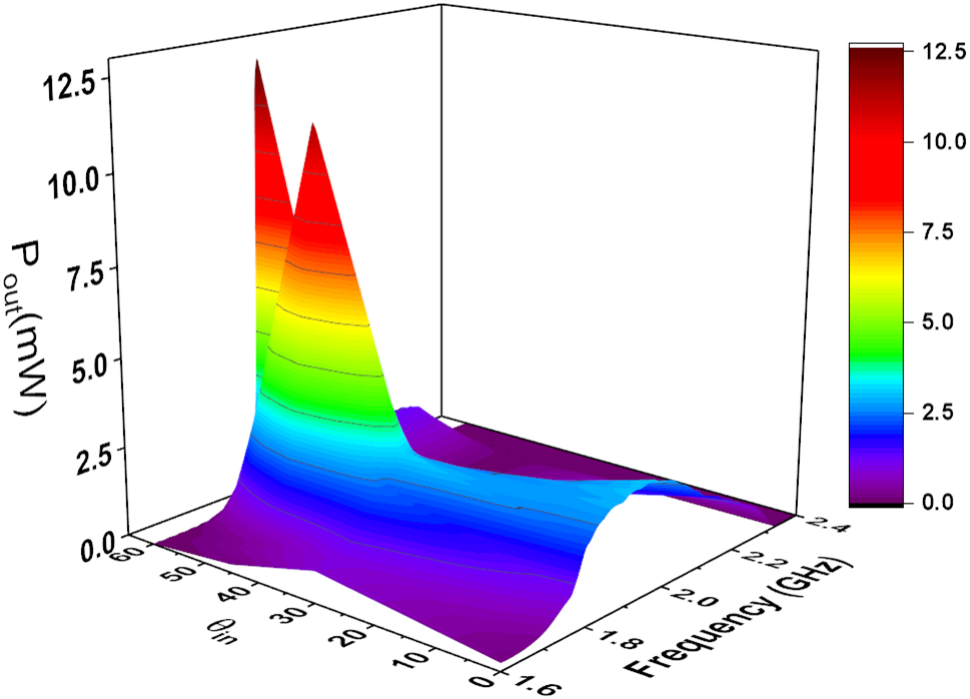
Simulated $P_{dc}$ for different angles of incidence and different frequencies. The power density of the incident field was kept constant at 1 W/m$^2$.

To demonstrate the effects of varying the incident angle on the output DC power, we illuminated the rectenna at different incident angles while keeping the power density fixed at 1 W/m$^2$. The variation in the incident angle does not affect the values of Thevenin impedance, however, it affects $V_{oc}$ for the same power density. To extract the $V_{oc}$ at different angles of incidence, the antenna was illuminated by a linearly polarized (in the y-direction) plane wave. The power density of the incident field was fixed at 1 W/m$^2$, and the angle of incidence $\theta$ was set to $30^{\circ }$, $45^{\circ }$ and $60^{\circ }$. The simulated $V_{oc}$ and output DC power at each angle of incidence is shown in Figs. 18 and 19. We observe a dramatic enhancement of the output DC power for an incident angle of $60^{\circ }$ over a narrow frequency range of 1.82 to 1.9 GHz. Similar behavior is observed for $45^{\circ }$ incidence, but over the narrow frequency range of 1.8 to 1.9 GHz. We emphasize that this enhancement is strictly dependent on the topology of the antenna used (TPA in this case). Other types of antennas may not give similar DC power profiles. These results, nevertheless, show the importance of providing full predictability of the rectenna system for all types of field excitation (polarization, power density, and angle of incidence). In fact, the simulation method presented in this work enables the production of a comprehensive graphical chart that shows the performance of the rectenna system under any excitation condition. Furthermore, we were able to create a 3D Figure using the provided method that simultaneously shows the variation of the output DC power with frequency and angle of incident, as shown in Fig.  20. Unexpectedly, the 3D figure enabled us to predict that the optimum angle of the incident at 2 GHz is 60$^o$. This wouldn’t be possible without this method.

The extracted values of $V_{oc}$ for each angle of incidence are loaded to the source of the circuit shown in Fig. 6. As depicted in the Figure, the variation of incident angle has a direct impact on the output DC power. The variation in the output DC power due to the change in incident angle is identical to that in $V_{oc}$.

## Conclusion

In this paper, we proposed a method to enable full simulation or characterization of a complete rectenna. This is accomplished by replacing the receiving antenna with its Thevenin equivalent circuit, and then using a circuit simulator after incorporating the equivalent circuit with the rectification circuit and load. To obtain the parameters of the Thevenin equivalent circuit, the CST full wave simulator was utilized to mimic the antenna in the receiving mode.

The primary objective of our method is to enable full simulation of the rectenna such that the designer can predict the accurate performance of the rectenna over the specified frequency range and for any angle of incidence and polarization. This can all be accomplished without fabrication or measurements. In fact, using our simulation method, one can produce a comprehensive graphical chart that shows the performance of a specific rectenna under any excitation condition (polarization, incidence angle, and power density). This feature enables the evaluation of the performance of any rectenna designed by a third party without resorting to measurements.

Another advantage of our method is its ability to optimize the rectification circuit for a specific receiving antenna to deliver maximum DC power. This advantage is critical since an optimal rectification circuit is strongly dependent on the particular antenna employed.

Finally, it is worth mentioning that some research investigated a multi-sine multiple-input multiple-output (MIMO) wireless power transfer (WPT) system to boost the output DC power, other studies focused on sending multi-sine signals with a high peak-to-average power ratio (PAPR), which has been shown to increase the efficiency of rectifier circuits’ RF-to-DC conversion^36–38^. This study, however, focused on CW excitation only.

## Data Availability

The datasets used and analyzed during the current study are available from the corresponding author (O.M.R.) on reasonable request.
